# Aqueous extracts of *Moringa oleifera* and *Carica papaya* moderately reduce gastrointestinal helminth burden in sheep under field conditions in Ghana

**DOI:** 10.3389/fvets.2026.1847798

**Published:** 2026-08-06

**Authors:** Prince Kyere Dwaah, Nana Yaa Awua-Boateng, Sylvia Afriyie Squire, Issah Bagulo, Denis Dekugmen Yar, Helen Djang-Fordjour, Helena Agyei Lartey

**Affiliations:** 1 Disease Investigation Farm/Regional Veterinary Laboratory, Veterinary Service Directorate, Techiman, Bono East, Ghana; 2 Department of Public Health Education, Faculty of Environment and Public Health Education, University of Skill Training and Entrepreneurial Development, Kumasi, Ghana; 3 Animal Health Division, Council for Scientific and Industrial Research (CSIR)—Animal Research Institute, CSIR College of Science and Technology, Accra, Greater Accra, Ghana; 4 Department of Animal Resources Development, CSIR College of Science and Technology, Accra, Ghana; 5 Department of Veterinary Nursing, School of Veterinary Sciences, University for Development Studies, Tamale, Ghana; 6 Department of Agriculture, Faculty of Applied Science and Technology, Sunyani Technical University, Sunyani, Ghana

**Keywords:** anthelmintic resistance, ethnoveterinary medicine, faecal egg count reduction, gastrointestinal helminths, sheep, sustainable livestock

## Abstract

**Introduction:**

Gastrointestinal helminth infections remain a major constraint to small ruminant production in tropical regions, and increasing reports of reduced anthelmintic efficacy are raising concerns for sustainable parasite control. This study evaluated the *in vivo* anthelmintic activity of aqueous extracts of *Moringa oleifera* leaves and *Carica papaya* seeds in sheep naturally infected under field conditions in Ghana.

**Methods:**

Thirty-three sheep were randomly assigned to four groups: *Moringa oleifera* extract (100 mg/mL), *Carica papaya* extract (100 mg/mL), albendazole (10%), and untreated control. Plant extracts were administered orally at 10 mL/kg body weight on Day 0, and faecal egg counts (FEC) were determined on Days 0, 5, 10, and 30 using the McMaster technique. Anthelmintic efficacy was assessed using the faecal egg count reduction test (FECRT).

**Results:**

At Day 30, *M. oleifera* achieved a 54.50% reduction in strongyle-type nematode egg counts, while *C. papaya* produced a 68.25% reduction. Albendazole showed lower efficacy (60.51%), whereas egg counts increased in the untreated control group. *C. papaya* achieved 95% of the benchmark of the World Association for the Advancement of Veterinary Parasitology on Day 30 against *Moniezia* spp. Although the interpretation is limited by the small sample size. No statistically significant differences were detected among treatment groups (*p* > 0.05). Sheep treated with *M. oleifera* showed the greatest numerical weight gain over the study period.

**Discussion:**

These findings indicate that aqueous extracts of *M. oleifera* and *C. papaya* exert moderate anthelmintic effects under field conditions but do not meet efficacy thresholds for standalone use. The reduced response to albendazole suggests possible resistance under local management conditions. Plant-based treatments may therefore serve as complementary tools within integrated parasite control strategies. Further studies incorporating larger sample sizes, standardized extraction protocols (including plant-to-solvent ratio, extraction time, extract concentration, and dosing regimen), and comprehensive phytochemical characterization (quantification of tannins, flavonoids, alkaloids, saponins, total phenolics, and proteolytic enzymes) are required to improve reproducibility and identify the compounds associated with anthelmintic activity.

**Figure:**
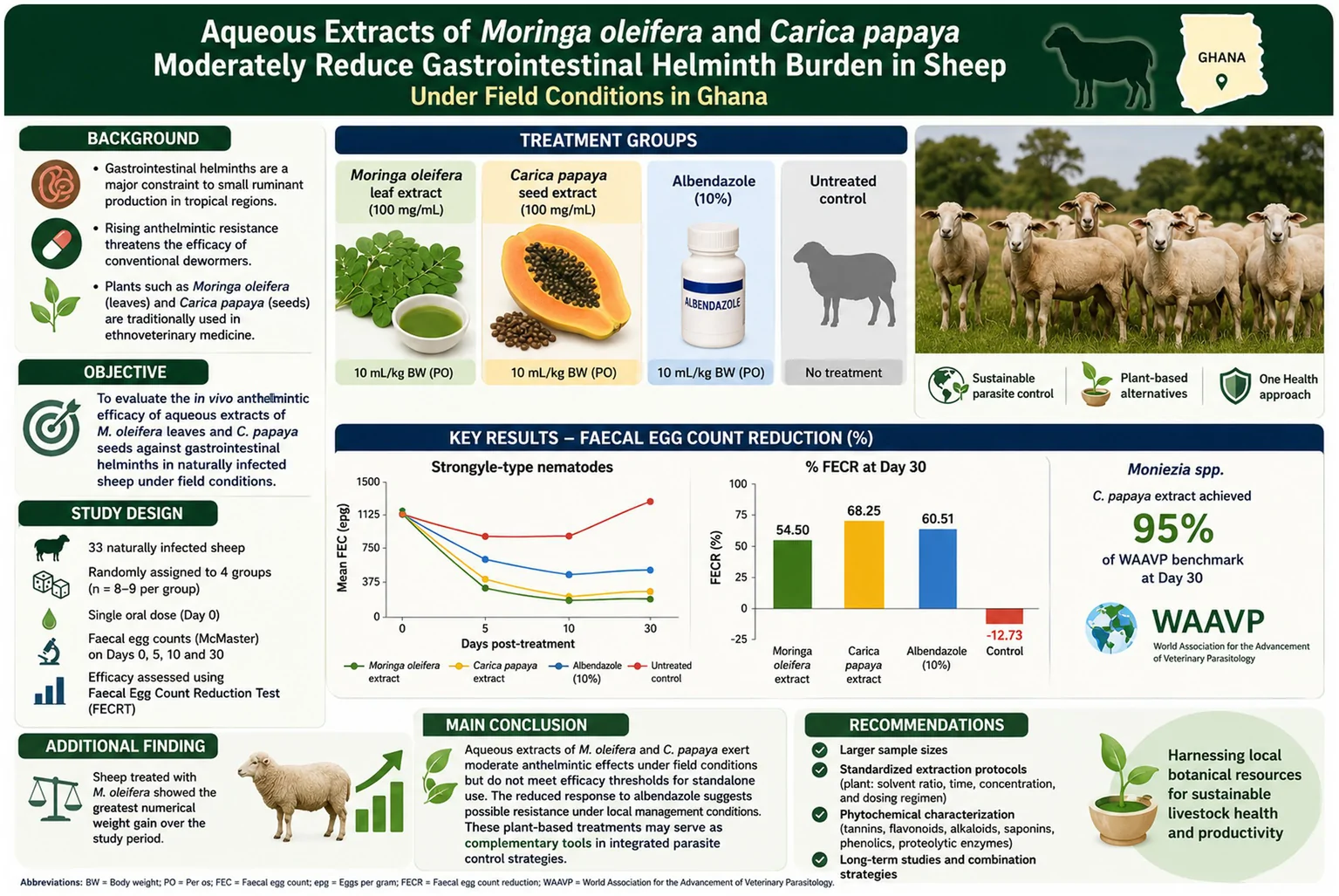

## Introduction

Small ruminant production remains central to livelihood systems across sub-Saharan Africa, including Ghana, where sheep contribute directly to household income, food security, and resilience in low-input farming environments (1, 2). In Ghana, the national sheep population exceeds 5 million, and gastrointestinal parasitism remains one of the most frequently reported constraints on productivity (3). Sheep production is an important source of meat, financial security, and socio-economic support, particularly in rural areas where livestock are often managed under extensive grazing systems. Despite their adaptability, productivity gains in these systems are frequently constrained by health-related challenges, among which gastrointestinal helminth infections are consistently identified as a major limiting factor (4).

Epidemiological studies from Ghana and other parts of West Africa have reported moderate to high prevalence of gastrointestinal helminth infections in small ruminants, with infection rates ranging from approximately 45% to over 70% depending on geographical location, season, management system, and diagnostic methods employed (5–9). These infections contribute to poor growth performance, reduced reproductive efficiency, decreased carcass yield, anaemia, and increased mortality, particularly among young and nutritionally stressed animals. The burden of helminthiasis is particularly pronounced in transitional forest-savannah and Guinea savannah agro-ecological zones, where climatic conditions favour the survival and transmission of infective parasite stages on pasture (9, 10).

Among gastrointestinal nematodes, strongyle-type nematodes *(predominantly Haemonchus* spp.*)* remain one of the most economically important parasites affecting sheep worldwide. Its significance is attributable to its high fecundity, rapid life cycle, blood-feeding behaviour, and remarkable capacity to adapt to environmental conditions, according to Kuiseu et al. (5), Opeto et al. (10) and Tinsley & Morgan (9). The parasite thrives under warm and humid tropical climates and is capable of causing severe anaemia, hypoproteinaemia, reduced weight gain, impaired productivity, and death in heavily infected animals (11). Studies conducted by Squire et al. (7), Kuiseu et al. (5) and Tinsley & Morgan (9) in West Africa have reported strongyle-type nematodes prevalence ranging from 40 to 75% among naturally grazing sheep, making it the dominant gastrointestinal nematode in many tropical production systems. Similar prevalence patterns have been reported by Opeto et al. (10) in ecological zones comparable to the present study area, highlighting the continuing importance of this parasite as a major constraint to sheep production.

In contrast, cestodes such as *Moniezia* spp. are generally regarded as less pathogenic than haematophagous nematodes. Nevertheless, their inclusion in the present study was considered important for several reasons. Mixed infections involving *Moniezia* spp. and gastrointestinal nematodes are common under extensive grazing conditions and may contribute cumulatively to reduced nutrient utilization, poor feed conversion efficiency, and impaired growth performance. Furthermore, *Moniezia* infections are frequently encountered during routine veterinary diagnostic investigations in Ghanaian small-ruminant production systems, according to the 2023 annual report from the Disease Investigation Farm and Regional Veterinary Laboratory. Evaluating activity against both nematode and cestode parasites therefore provides a broader assessment of the antiparasitic spectrum of the investigated plant extracts and enhances the practical relevance of the findings.

For decades, the control of gastrointestinal helminths has relied primarily on repeated administration of broad-spectrum anthelmintic drugs. Although these compounds initially improved animal health and productivity, their long-term effectiveness has become increasingly threatened by the emergence and spread of anthelmintic resistance. Reports of reduced efficacy of benzimidazoles, macrocyclic lactones, and imidazothiazoles have been documented across many regions of the world, including Africa, raising concerns regarding the sustainability of current parasite control strategies (12, 13). In many smallholder production systems, frequent blanket deworming, under-dosing, improper drug selection, and limited access to diagnostic monitoring further accelerate the development of resistant parasite populations (14).

The growing threat of anthelmintic resistance has stimulated interest in integrated parasite management (IPM) approaches. Integrated parasite management refers to the strategic combination of multiple complementary interventions designed to reduce parasite burdens, minimize reliance on chemical anthelmintics, and slow the development of resistance. Core components of IPM include targeted selective treatment based on diagnostic evidence, rotational grazing, nutritional supplementation, genetic selection for parasite-resistant animals, biological control measures, routine surveillance using faecal egg count monitoring, and the incorporation of validated ethnoveterinary remedies (11, 14). Such integrated approaches are increasingly recognized as essential for achieving sustainable parasite control in livestock systems.

Within this framework, ethnoveterinary medicine has gained renewed attention as a potentially sustainable and locally accessible component of parasite management. Livestock keepers have long used medicinal plants for the treatment and prevention of parasitic diseases, particularly in resource-limited settings where access to conventional veterinary services may be constrained (15). Plant-derived compounds may offer several advantages, including local availability, affordability, environmental compatibility, and a reduced likelihood of resistance development due to their chemically diverse mixtures of bioactive constituents (16). However, despite increasing interest in botanical anthelmintics, their practical application remains limited by variability in efficacy, lack of standardized formulations, and insufficient validation under field conditions (17).

Beyond their direct antiparasitic properties, medicinal plants may also influence host immunity and gastrointestinal health through interactions with the gut microbiota. Bioactive compounds such as polyphenols, flavonoids, tannins, and plant polysaccharides have been shown to modulate microbial communities, enhance intestinal barrier integrity, and regulate immune responses within the gastrointestinal tract. Recent evidence indicates that these interactions can strengthen mucosal defence mechanisms, improve resistance to gastrointestinal pathogens, and promote host resilience during parasitic infections (18). Such microbiota-mediated immunological effects may complement direct antiparasitic activity and contribute to overall improvements in animal health and productivity.

Plant polysaccharides may further modify gastrointestinal ecology through microbial fermentation processes. Fermentation of these compounds by beneficial gut microorganisms generates metabolites such as short-chain fatty acids, which influence intestinal pH, epithelial integrity, nutrient utilization, and microbial community structure. These microbiota-driven changes may indirectly affect parasite establishment, survival, fecundity, and host tolerance to infection. Li et al. (19) demonstrated that dietary polysaccharides can significantly alter gut microbial composition and metabolic activity, highlighting the importance of microbial-mediated pathways in maintaining gastrointestinal health and potentially influencing host–parasite interactions.

*Moringa oleifera (M. oleifera)* and *Carica papaya (C. papaya)* are among the most widely distributed medicinal plants in tropical regions and are commonly employed in traditional livestock healthcare. Both species contain numerous bioactive compounds, including tannins, flavonoids, alkaloids, saponins, phenolics, and proteolytic enzymes that may interfere with parasite survival, development, metabolism, and reproduction (20, 21). Although promising results have been reported from laboratory investigations and controlled experimental studies, much of the available evidence has been generated using solvent-based extracts under highly controlled conditions. Consequently, the extent to which these findings translate into practical effectiveness under natural grazing conditions remains uncertain.

A major knowledge gap, therefore, exists regarding the field performance of aqueous plant preparations that can be readily prepared and applied by smallholder farmers. Evaluations conducted under natural grazing conditions are essential for determining the real-world efficacy of botanical anthelmintics and their potential contribution to sustainable parasite management programmes. Comparative assessment against commonly used synthetic anthelmintics is also important for contextualizing efficacy and identifying practical applications within livestock production systems.

The present study was designed to address these knowledge gaps by evaluating the *in vivo* anthelmintic activity of aqueous extracts of *M. oleifera* leaves and *C. papaya* seeds in sheep naturally infected with gastrointestinal helminths under field conditions in Ghana.

### Hypothesis

This study hypothesized that aqueous extracts of *M. oleifera* leaves and *C. papaya* seeds would significantly reduce faecal egg counts of *strongyle-type nematodes* and *Moniezia* spp. in naturally infected sheep under field conditions and would provide complementary antiparasitic effects comparable to those achieved by conventional albendazole treatment. It was further hypothesized that animals receiving plant-based treatments would demonstrate improved productivity indicators, particularly body weight gain, compared with untreated controls.

## Materials and methods

### Study area

The study was conducted at the Disease Investigation Farm (DIF) in Techiman, Bono East Region, Ghana, an area known for its high prevalence of gastrointestinal parasitic infections in small ruminants due to the clinic and laboratory attached to it, to investigate conventional dewormers by the Veterinary Directorate Service (VSD) of Ghana. The site lies within the transitional ecological zone (7.59529°N, −1.93331°W), characterized by Guinea savannah and semi-deciduous vegetation. Techiman experiences a tropical savannah climate with annual rainfall of 1,200–1,500 mm (3, 22) and temperatures ranging from 22 °C to 34 °C during the study period [September–October 2024].

### Plant material collection and botanical identification

*C. papaya* seeds and *M. oleifera* leaves were selected based on ethnoveterinary practices reported by small ruminant farmers in the Techiman area of the Bono East Region, Ghana. Formal consultations with farmers and local animal health workers indicated that these plant materials are commonly administered as traditional remedies for the management of gastrointestinal parasitic infections in sheep and goats by the research team. The selection was further supported by previous studies that have reported potential anthelmintic properties of *C. papaya* seeds and *M. oleifera* leaves in small ruminants and other livestock species (16, 23, 24).

Mature *C. papaya* fruits and fresh *M. oleifera* leaves were harvested from organically maintained plants within DIF. Voucher specimens were prepared and deposited in the institutional herbarium under accession numbers CP-DIF-2024-01 (*Carica papaya*) and MO-DIF-2024-02 (*Moringa oleifera*) to ensure proper documentation and traceability of the plant materials used in this study.

### Plant materials and preparation of extracts

The harvested plant materials were washed with distilled water to remove debris (25) and air-dried under shade at ambient temperature (25–30 °C) for 10–14 days until constant weight was achieved (26, 43). The dried materials were ground using a laboratory mill and sieved through a 1-mm mesh to obtain uniform powders.

Aqueous extracts were prepared by maceration following established procedures with minor modifications (16, 17, 23, 24, 27). Briefly, 50 g of powdered plant material were mixed with 500 mL of distilled water (plant-to-solvent ratio 1:10 w/v) in sterile glass containers. The mixtures were maintained under continuous agitation using a laboratory shaker at room temperature (25 °C) for 12 h. The macerates were subsequently filtered through sterile muslin cloth, followed by filtration through Whatman No. 1 filter paper to remove particulate matter.

The filtrates were considered the crude aqueous extracts. Extract yield was calculated as the percentage of dry extract obtained relative to the initial plant powder weight after evaporation of a measured aliquot at 40 °C to constant weight. The resulting concentrations were expressed in mg/mL of crude extract. Fresh extracts were prepared every three days during the experimental period and stored in sterile amber bottles at 4 °C for a maximum of 72 h prior to administration to minimize degradation and potential batch variability.

### Preliminary phytochemical evidence

Previous phytochemical investigations of *Carica papaya* seeds and *Moringa oleifera* leaves have identified several bioactive secondary metabolites that may contribute to their anthelmintic activity, including tannins, flavonoids, saponins, alkaloids, phenolic compounds, and proteolytic enzymes (16, 23, 24). Following extraction, the concentration of each crude aqueous extract was determined gravimetrically after evaporation of a measured aliquot to constant weight and expressed as mg/mL of crude extract. Sheep received the extracts orally at a dose of 10 mL/kg body weight, corresponding to the crude extract concentration obtained from the extraction process. Because the specific bioactive constituents responsible for the observed anthelmintic effects were not isolated or quantified in the present study, doses are reported as crude extract equivalents rather than concentrations of individual active compounds. Future studies incorporating phytochemical characterization and quantification of active metabolites will enable more precise dose standardization.

### Determination of safe dosage (pilot tolerability trial)

A preliminary dose-ranging tolerability trial was conducted prior to the main experiment to establish a safe dosage level for oral administration of the plant extracts. Ten clinically healthy sheep that were not included in the main experimental groups were used for the pilot assessment. Five animals each were randomly assigned to receive aqueous extracts of either *C. papaya* seeds or *M. oleifera* leaves at doses ranging from 1 to 10 mL/kg body weight.

Following administration, animals were monitored for 72 h for clinical signs of toxicity. Observed parameters included feed intake, water consumption, faecal consistency, body temperature, respiratory rate, heart rate, and general behaviour. Additional observations included the presence of neurological signs, gastrointestinal disturbances, lethargy, or abnormal locomotion.

No adverse clinical signs or behavioural abnormalities were observed at any tested dose level during the monitoring period. Consequently, the highest tested dose (10 mL/kg body weight) was selected for the efficacy trial. The selection of this dose was based on the primary objective of the pilot study, which was to establish short-term safety and determine whether the extracts possessed measurable anthelmintic activity under field conditions. A formal dose–response evaluation was not undertaken because of limitations in animal availability, logistical constraints, and the exploratory nature of the study. The highest tolerated dose was therefore chosen to maximize the likelihood of detecting a biological effect while remaining within a clinically safe range. Nevertheless, future investigations should evaluate multiple dose levels to establish dose–response relationships and identify the optimum therapeutic dosage for each plant extract.

### Experimental animals and management

Thirty-three naturally infected sheep maintained at the DIF were used in the study. Animals included both sexes and different age categories, reflecting the existing farm population. Before inclusion, all animals underwent a clinical examination to ensure general health status. The study included only 8–9 animals per treatment group, which is below the sample size generally recommended for robust Faecal Egg Count Reduction Test (FECRT) interpretation under World Association for the Advancement of Veterinary Parasitology (WAAVP) guidelines. Consequently, confidence intervals around efficacy estimates are likely to be wide, and treatment differences should be interpreted cautiously.

Inclusion criteria consisted of:

- Natural infection with gastrointestinal helminths confirmed by faecal egg counts (FEC) with a load of more than 200 eggs per gram [EPG] (12, 27),
- Absence of anthelmintic treatment within the preceding eight weeks, and
- A body condition score considered adequate for participation in the trial.

Animals were housed under standard management conditions and fed natural pasture supplemented with crop residues, while clean water was provided ad libitum. A 7-day acclimatization period was allowed before the commencement of the experiment.

### Experimental design and randomization

A randomized controlled experimental design was used. The 33 sheep were allocated to four treatment groups. Randomization was performed using a computer-generated random number table. To minimize baseline variability, group allocation considered initial FEC and body weight to achieve approximate balance among treatment groups.

The final distribution consisted of eight animals each in Groups 2–4 and nine animals in Group 1. Although the population included animals of different ages and sexes, allocation aimed to distribute these characteristics as evenly as possible across groups.

Albendazole was selected as the positive control because it is the most commonly used conventional anthelmintic in the Techiman area, according to local veterinary officers, animal health workers, and sheep farmers. The commercial formulation used in this study was albendazole 10% oral suspension. Albendazole was administered at a dose of 10 mg/kg body weight in accordance with the manufacturer’s recommendations and internationally recognized guidelines for the treatment of gastrointestinal helminth infections in sheep (14, 28). This dosage represents the standard therapeutic dose routinely used in veterinary practice and was selected to provide an appropriate benchmark against which the efficacy of the plant extracts could be compared. The use of the recommended therapeutic dose also ensured that any observed reduction in efficacy was less likely to be attributable to under-dosing and more reflective of parasite susceptibility under field conditions. Treatments and dosages administered to each group are presented in Table 1.

**Table 1:** Experimental groups, treatments, and administered dosages in naturally infected sheep.

Group	Treatment	Dosage
1	Distilled water (negative control)	10 mL/kg BW
2	*Carica papaya* seed extract	10 mL/kg BW
3	*Moringa oleifera* leaf extract	10 mL/kg BW
4	Albendazole (positive control)	10 mg/kg BW

BW, body weight.

All treatments were administered orally on Day 0 using calibrated dosing syringes. Faecal samples were collected on Days 0, 5, 10, and 30 for parasitological evaluation.

### Faecal collection and parasitological examination

Faecal samples were collected directly from the rectum of each animal using disposable gloves following standard operating procedures (14). Samples were placed in labelled sterile containers and transported immediately to the laboratory for analysis.

Quantitative FEC were performed using the McMaster flotation technique (28–31). Briefly, 3 g of faeces were homogenized in 60 mL of saturated sodium chloride solution and filtered through a sieve to remove debris. The filtrate was transferred into McMaster counting chambers and examined under a light microscope at ×10 magnification. The number of helminth eggs within the calibrated grid was recorded and expressed as EPG of faeces.

Strongyle-type eggs were identified morphologically during faecal examination. Because species-level differentiation of strongyle eggs is not possible using the McMaster technique alone, the presence of *H. contortus* was inferred from previous epidemiological reports indicating its predominance in small ruminants in Ghana. No larval culture or molecular confirmation was performed.

To reduce observational bias, laboratory personnel performing the egg counts were blinded to treatment allocation.

### Anthelmintic efficacy assessment

Anthelmintic efficacy was evaluated using the FECRT following the guidelines described by Denwood et al. (30), Kaplan et al. (14) and Coles et al. (28). The percentage reduction in FEC was calculated as: FECRT = 100 x $(\;1-\frac{T_{2}}{T_{1}}$ x $\frac{C_{1}}{C_{2}}$ ), where; $T_{1}$ = arithmetic mean FEC of the treated group before treatment, $T_{2}$ = arithmetic mean FEC of the treated group after treatment, $C_{1}$ = arithmetic mean FEC of untreated control before treatment and $C_{2}$ = arithmetic mean FEC of untreated control after treatment.

### Statistical analysis

Descriptive statistics were expressed as mean ± standard deviation (SD). Data were initially explored for normality using the Shapiro–Wilk test.

Differences in mean FEC among treatment groups were evaluated using one-way analysis of variance (ANOVA), followed by Tukey’s Honest Significant Difference (HSD) test for pairwise comparisons when significant differences were detected (*p* < 0.05). Associations between the infection type were evaluated using Fisher’s Exact Test. Paired *t*-tests were used to compare pre and post-treatment FEC within each group.

All statistical analyses were performed using Microsoft Excel 2019 and appropriate statistical add-ins. A *post hoc* power analysis (*α* = 0.05) indicated that the study had statistical power greater than 0.80 to detect treatment effects.

## Results

### Baseline helminth infection and faecal egg counts

At baseline (Day 0), gastrointestinal helminth infection was detected in all 33 sheep included in the study. Mixed infections involving strongyle-type nematodes and *Moniezia* spp. were the most prevalent, occurring in 60.6% (20/33) of animals, while single infections with strongyle-type nematodes and *Moniezia* spp. accounted for 30.3% (10/33) and 9.1% (3/33) of infections, respectively (Table 2). The distribution of infection types across sex and age categories was evaluated using Fisher’s Exact Test because of small subgroup sizes. No significant association was detected between animal category and infection type (*p* = 0.421), indicating that the observed infection patterns were comparable across the study population. Associations between infection type (*H. contortus* only, *Moniezia* spp. only, and mixed infection) and animal sex/age category were evaluated using Fisher’s Exact Test because several contingency table cells contained expected counts below five. Statistical significance was declared at *p* < 0.05.

**Table 2:** Distribution of pre-treatment helminth infection types according to sex and age category (*n* = 33 sheep).

Sex/age category	No. of sheep	Strongyle-type nematodes only *n* (%)	Moniezia spp. only *n* (%)	*n* (%)
Ram	4	0 (0.0)	0 (0.0)	4 (100.0)
Ewe	21	8 (38.1)	2 (9.5)	11 (52.4)
Young Ram	4	2 (50.0)	0 (0.0)	2 (50.0)
Young Ewe	4	0 (0.0)	1 (25.0)	3 (75.0)
Total	33	10 (30.3)	3 (9.1)	20 (60.6)

Fisher’s exact test: *p* = 0.421.

1. Both moniezia expansa and moniezia benedeni were grouped as moniezia spp.

2. Due to small expected cell frequencies, fisher’s exact Test was used instead of Pearson’s chi-square test.

3. No statistically significant association was observed between sex/age category and infection type (Fisher’s exact test, *p* = 0.421).

### Anthelmintic efficacy against *H. contortus*

Table 3 showed that by Day 30 post-treatment, sheep receiving aqueous *Moringa oleifera* leaf extract exhibited a mean FECRT of 54.50% (95% not reliably estimable) against strongyle-type nematodes. Treatment with aqueous *Carica papaya* seed extract resulted in a mean reduction of 68.25% (95% CI: 55.49–81.80). In contrast, the conventional anthelmintic (albendazole) produced a mean FECRT of 60.51% (95% CI: 32.89–81.02). The untreated control group showed an increase in mean FEC over the same period.

**Table 3:** WAAVP-controlled FECRT results against Strongyle-type nematodes.

Treatment		Days				
	0 Mean ± SD	10 Mean ± SD	30 Mean ± SD	FECRT % (Day 10)	FECRT % (Day 30)	95% CI
AML	668.75 ± 152.6^c^	250.0 ± 65.5^ab^	37.5 ± 18.3^a^	42.44	54.50	Not reliably estimable*
APS	718.75 ± 111.8^b^	431.3 ± 89.1^ab^	281.3 ± 86.5^a^	48.67	68.25	55.49–81.80
A 10%	706.25 ± 124.8	550.0 ± 130.6	343.8 ± 151.0	33.38	60.51	32.89–81.02
D. water	788.9 ± 144.0	922.2 ± 163.3	972.2 ± 164.4	Reference control	Reference control	Reference control

1. Values with different superscripts in the same row are statistically different.

2. “AML” is aqueous Moringa leaves, “APS” is aqueous papaya seeds, “A 10%” is albendazole 10% and “D. water” is distilled water.

3. *Bootstrap confidence interval estimation was unstable because of zero-count observations in the dataset.

### Anthelmintic response against *Moniezia* spp

Among animals receiving aqueous *Moringa oleifera* leaf extract, the mean FECRT against *Moniezia* spp. was 68.88% at Day 10, increasing to 81.91% (65.63–91.33) by Day 30. Sheep treated with aqueous *Carica papaya* seed extract exhibited mean reductions of 77.63% at Day 10 and 97.14% at Day 30, as shown in Table 4.

**Table 4:** WAAVP-controlled FECRT results against Moniezia spp.

Treatment		Days				
	0 Mean ± SD	10 Mean ± SD	30 Mean ± SD	FECRT % (Day 10)	FECRT % (Day 30)	95% CI
AML	262.5 ± 63.2^c^	100.0 ± 42.3^ab^	25.0 ± 16.4^a^	68.88	81.91	65.63–91.33
APS	237.5 ± 82.2	112.5 ± 39.8	18.8 ± 9.1	77.63	97.14	Not reliably estimable*
A 10%	106.3 ± 46.7	68.8 ± 26.6	31.3 ± 16.2	69.44	93.62	Not reliably estimable*
D. water	94.4 ± 32.7	200.0 ± 55.9	261.1 ± 66.0	Reference Control	Reference Control	Reference Control

1. Values with different superscripts in the same row are statistically different.

2. “AML” is aqueous Moringa leaves, “APS” is aqueous papaya seeds, “A 10%” is albendazole 10% and “D. water” is distilled water.

3. *Bootstrap confidence interval estimation was unstable because several animals had zero egg counts.

### Effect of treatment and time on anthelmintic efficacy

ANOVA was conducted to evaluate the effects of treatment type, time, and their interaction on faecal egg count reduction percentages *(*Table 5*).* No statistically significant main effects of treatment (*p* = 0.219) or time (*p* = 0.117) were detected. Similarly, the interaction between treatment and time was not significant (*p* = 0.995), indicating that reductions in FEC over the experimental period did not differ statistically among treatment groups.

**Table 5:** ANOVA assessing the effects of treatment, time, and their interaction on FECRT (%).

Source of variation	Sum of squares	df	*F*-statistic	*p*-Value
Treatment type	2276.78	2	1.8	0.219
Time	3468.11	2	2.75	0.117
Treatment × time (interaction)	115.22	4	0.046	0.995
Residual (error)	5677.5	9		

Interpretation: the lack of significant interaction (*p* = 0.995) implies a consistent treatment effect over time.

### Weight gain change

Mean body weight increased over time in all treated groups (Table 6). Sheep treated with aqueous *M. oleifera* leaf extract exhibited the greatest numerical increase in mean body weight over the 30 days from 24.5 ± 2.9 to 29.19 ± 3.1making the mean gain 4.7 kg, followed by those treated with aqueous *C. papaya* seed extract and Conventional dewormer. Sheep in the untreated control group showed minimal change in body weight over the same period. One-way ANOVA did not detect statistically significant differences in body weight among treatment groups at any sampling point (*p* > 0.05).

**Table 6:** Mean body weight (kg, mean ± SD) of sheep by treatment group.

Treatment		Days			*P*-value
	0	5	10	30	
Albendazole 10%	24.13 ± 2.5	25.06 ± 2.5	26.13 ± 2.4	27.63 ± 2.4	0.770
Aqueous moringa leaves	24.5 ± 2.9	25.63 ± 3.0	27.06 ± 3.1	29.19 ± 3.1	0.720
Aqueous papaya seeds	26 ± 2.9	27.06 ± 2.8	28.06 ± 2.8	29.06 ± 2.9	0.886
Distilled water	26 ± 2.6	25.44 ± 2.5	26.22 ± 2.7	26.39 ± 2.8	0.995

No significant differences in body weight were detected among treatment groups (one-way ANOVA; *p* > 0.05).

## Discussion

The present study evaluated the *in vivo* anthelmintic activity of aqueous extracts of *Moringa oleifera* leaves and *Carica papaya* seeds in sheep naturally infected with gastrointestinal helminths under field conditions in Ghana. Baseline infection patterns were dominated by mixed Strongyle-type nematodes -*Moniezia* spp. infections (60.6%), although infection type did not differ significantly among sex and age categories (Fisher’s exact test, *p* = 0.421). This suggests that exposure to gastrointestinal helminths was relatively uniform across the study population and that subsequent treatment responses were unlikely to be confounded by demographic differences in the distribution of infections.

According to WAAVP guidelines, an anthelmintic is generally considered effective when the faecal egg count reduction test (FECRT) exceeds 95%, with lower values suggesting reduced efficacy or possible resistance depending on accompanying confidence intervals (14). In the present study, aqueous *C. papaya* achieved an FECRT of 97.14% against *Moniezia* spp. at Day 30, approaching the WAAVP efficacy threshold, while albendazole achieved 93.62 and 81.91% for *M. oleifera*. Against strongyle-type nematodes, FECRT values reached 68.25% for *C. papaya*, 60.51% for albendazole and 54.50 for *M. oleifera* by Day 30. Although these reductions indicate substantial antiparasitic activity, most, except *C. papaya* against *Moniezia* spp. consistently achieved the ≥95% threshold required for classification as highly effective stand-alone anthelmintic interventions. The findings therefore support the interpretation that the plant extracts possess biologically relevant activity but should presently be regarded as complementary rather than replacement therapies (11, 17).

The efficacy observed for *M. oleifera* against strongyle-type nematodes is broadly consistent with previous *in vivo* and experimental investigations. Páez-León et al. (21) demonstrated that *M. oleifera* leaf extracts exert direct nematicidal effects against strongyle-type nematodes, resulting in significant reductions in larval viability and development. Similarly, reviews of botanical parasite control have documented moderate to high reductions in gastrointestinal nematode burdens following administration of phytochemical-rich plant materials, particularly those containing condensed tannins, flavonoids, and phenolic compounds (11, 32). The FECRT value of 94.39% recorded in the present study is notably higher than that of many field-based evaluations of crude botanical preparations, suggesting that *M. oleifera* may possess practical value under natural grazing conditions despite the challenges imposed by continual reinfection pressure.

The response observed with *C. papaya* was comparatively lower against strongyle-type nematodes but remained substantial. Previous studies have attributed the antiparasitic activity of *C. papaya* seeds primarily to proteolytic enzymes such as papain and chymopapain, together with alkaloids, benzyl isothiocyanate, flavonoids, and tannins that can damage parasite cuticles, disrupt metabolic processes, and impair egg production (16, 20). The gradual decline in egg counts observed in the present study is consistent with mechanisms affecting parasite fecundity and survival rather than immediate parasite elimination. However, because neither phytochemical profiling nor parasite recovery studies were performed, the specific pathways responsible for the observed reductions remain unconfirmed.

An important finding was the comparatively poor performance of albendazole. The FECRT value of 51.32% against strongyle-type nematodes falls substantially below the efficacy levels expected for susceptible parasite populations and is well below the WAAVP threshold for effective treatment (14). Similar reductions have been reported in regions where prolonged reliance on benzimidazole compounds has contributed to the emergence of anthelmintic resistance (4, 13). Although resistance was not formally confirmed in the present study through larval culture, molecular diagnostics, or egg hatch assays, the observed reduction suggests diminished parasite susceptibility under local field conditions. Consequently, the superior numerical performance of *M. oleifera* relative to albendazole should not be interpreted as evidence that the plant extract is intrinsically more potent than a conventional anthelmintic. Rather, it indicates that botanical preparations may retain useful antiparasitic activity in production systems where the effectiveness of established drugs has declined.

The mechanisms underlying the observed antiparasitic effects are likely multifactorial. Tannins have been shown to bind parasite cuticular proteins and interfere with nutrient acquisition, while flavonoids and phenolic compounds may induce oxidative stress and disrupt energy metabolism within helminths (16, 25). Experimental evidence further suggests that phytochemicals derived from *M. oleifera* can impair larval development and reduce parasite viability (21). However, the present study did not quantify individual bioactive compounds or assess their concentrations within the administered extracts. Therefore, mechanistic explanations should be interpreted cautiously and viewed as biologically plausible rather than experimentally demonstrated outcomes of the current work.

Beyond direct antiparasitic activity, bioactive plant compounds may influence host immunity. Hassan et al. (33) demonstrated that plant-derived bioactive constituents can regulate innate and adaptive immune responses through modulation of cytokine production, inflammatory signalling pathways, and immune-cell activity. Such immunomodulatory effects may enhance host resilience to parasitic infections by improving parasite tolerance and facilitating more effective immune-mediated control. The greater numerical weight gain observed in sheep treated with *M. oleifera* may therefore reflect not only reductions in parasite burden but also improved physiological resilience associated with immune and metabolic support. Nevertheless, immune parameters were not measured in the present study; therefore, any contribution of immunomodulation remains inferential and requires confirmation through future investigations incorporating cytokine profiling, haematological biomarkers, and cellular immune assessments.

The observed increases in body weight require careful interpretation. *M. oleifera* is recognized as a highly nutritious forage resource containing substantial concentrations of protein, vitamins, minerals, and antioxidant compounds (34, 35). Consequently, improved growth performance cannot be attributed solely to anthelmintic activity. Reduced parasite burden may have enhanced nutrient utilization and feed efficiency, while the nutritional composition of the plant itself may have independently supported growth and recovery. The present study design does not permit separation of these two effects. Therefore, the body weight responses should be interpreted as reflecting the combined influence of nutritional supplementation and parasite control rather than evidence of a direct anthelmintic-growth relationship.

The higher reductions were observed against *Moniezia* spp. compared with strongyle-type nematodes suggest possible parasite-specific differences in susceptibility. Similar variability has been reported in previous studies evaluating botanical anthelmintics, where efficacy frequently differs among parasite taxa due to variations in physiology, feeding behaviour, and metabolic pathways (11). However, because only three animals harboured single *Moniezia* infections, conclusions regarding cestode-specific efficacy should be considered preliminary.

Several limitations should be considered when interpreting these findings. Albendazole produced lower FECRT values than expected for fully effective treatment. However, the present study was not designed to diagnose anthelmintic resistance. Confirmation of resistance would require additional investigations such as larval culture, species differentiation, molecular detection of resistance-associated mutations, repeated FECRT assessments, and verification of product quality and dosing accuracy. Therefore, the observed efficacy should be interpreted only as reduced treatment performance under the conditions of this study. The relatively small sample size and short study duration may have limited statistical power to detect treatment differences. The use of crude aqueous extracts without phytochemical standardization prevents identification of the active constituents responsible for efficacy and may contribute to batch-to-batch variability. Furthermore, no assessment of gut microbiota composition, intestinal barrier integrity, inflammatory markers, or mucosal immune responses was undertaken. Recent evidence indicates that plant bioactives can exert important antiparasitic and health-promoting effects through modulation of host-microbiota interactions and enhancement of intestinal barrier function (36, 37). The absence of such measurements restricts interpretation of whether the observed reductions in egg counts resulted solely from direct parasite toxicity or were partially mediated through microbiota-associated and host-mediated mechanisms. Future studies integrating parasitological, phytochemical, immunological, and microbiome analyses would provide a more comprehensive understanding of the pathways underlying botanical anthelmintic activity.

Overall, the findings demonstrate that aqueous preparations of *M. oleifera* and *C. papaya* possess measurable *in vivo* antiparasitic activity under natural grazing conditions. While the efficacy achieved did not consistently satisfy internationally accepted thresholds for stand-alone anthelmintic use, the results support their potential role as complementary components of integrated parasite management programmes, particularly in settings where reduced susceptibility to conventional anthelmintics may compromise parasite control.

### Novelty and contribution

This study contributes to the existing body of knowledge by providing field-based evidence on the anthelmintic activity of *M. oleifera* and *C. papaya* using aqueous preparations that reflect practical on-farm application in smallholder systems. Unlike many previous investigations conducted under controlled laboratory conditions or using solvent extracts, the present work evaluates these plants under natural grazing conditions where reinfection pressure is present. In addition, the study offers a direct comparison with a commonly used synthetic anthelmintic, thereby providing context for interpreting efficacy in a setting where reduced drug performance may indicate emerging resistance. By integrating parasitological outcomes with observations on animal performance, this work highlights the potential role of locally available plant resources as complementary tools within integrated parasite management strategies in tropical livestock systems.

## Conclusion

Aqueous extracts of *M. oleifera* leaves and *C. papaya* seeds demonstrated measurable *in vivo* anthelmintic activity against gastrointestinal helminths in naturally infected sheep under field conditions, with *M. oleifera* showing the greatest reduction in faecal egg counts and associated improvement in body weight. Although neither botanical treatment achieved the efficacy threshold required for stand-alone parasite control, their performance relative to albendazole suggests potential value as complementary components of integrated parasite management strategies in smallholder production systems. Interpretation of these findings is limited by the small sample size, single-site design, and use of non-standardized crude extracts. Future studies should include larger multi-location trials, phytochemical standardization, and dose–response evaluations to establish the practical applicability of these botanicals for sustainable helminth control.

## Data Availability

The dataset supporting the findings of this study is publicly available and can be accessed at the following repository (38), https://doi.org/10.17632/rjyxybj2hz.
